# Mitochondrial genetic background in Ghanaian patients with primary open-angle glaucoma

**Published:** 2012-07-18

**Authors:** Khaled K. Abu-Amero, Michael A. Hauser, Gamal Mohamed, Yutao Liu, Jason Gibson, Ana M. Gonzalez, Stephen Akafo, R. Rand Allingham

**Affiliations:** 1 Ophthalmic Genetics Laboratory, Department of Ophthalmology, College of Medicine, King Saud University, Riyadh, Saudi Arabia; 2 Center for Human Genetics, Duke University Medical Center, Durham, NC; 3 Liverpool School of Tropical Medicine, Pembroke Place, Liverpool, U.K; 4 Área de Genética, Departamento de Parasitología, Ecología y Genética, Facultad de Biología, Universidad de La Laguna (ULL), La Laguna (Tenerife), Spain; 5 Department of Ophthalmology, University of Ghana, Accra, Ghana; 6 Department of Ophthalmology, Duke University Eye Center, Durham, NC; 7 Department of Ophthalmology, College of Medicine, University of Florida, Jacksonville, FL

## Abstract

**Purpose:**

Prevalence rates for primary open-angle glaucoma (POAG) are significantly higher in Africans than in European or Asians. It has been reported recently that mitochondrial DNA (mtDNA) lineages of African origin, excluding L2, conferred susceptibility to POAG in Saudi Arabia. This prompted us to test the role of mtDNA haplogroups in the incidence of POAG in the Ghanaian population who has a high frequency of L2 lineages.

**Methods:**

DNA was extracted from two independent cohorts of clinically diagnosed POAG patients (n=373) and healthy controls (n=451). All patients and controls were from Accra and Tema (the southern region of Ghana). The hypervariable region-I (HVS-I) and coding regions comprising mtDNA haplogroup diagnostic polymorphisms were polymerase chain reaction (PCR) amplified and sequenced in all patients and controls and the haplotypes obtained were assorted into haplogroups and their frequencies compared between cohorts.

**Results:**

No statistically significant differences were found in mtDNA haplogroup frequencies between POAG patients and matched controls in this cohort for the various mtDNA haplogroups tested.

**Conclusions:**

In this Ghanaian cohort, mtDNA haplogroups do not seem to confer susceptibility to POAG.

## Introduction

Primary open-angle Glaucoma (POAG) is the main cause of irreversible blindness worldwide [1]. Heritability estimates evidenced that POAG has a strong genetic component [2]. In fact, linkage analysis, case-control and genome-wide association approaches have related more than 20 nuclear genetic loci to POAG [3]. However, these associations only account for about 5% of POAG cases, reinforcing the idea that POAG is a complex multifactorial disorder result of the interaction of multiple genes and environmental factors [4]. In addition, several epidemiological studies have shown that a positive maternal family history significantly increases the risk of developing POAG than a positive paternal family history [5-7] raising the possibility of an involvement of maternal cytoplasmic factors, as the mitochondria, in the transmission and pathogenesis of POAG. A characteristic of the haploid mitochondrial genome (mtDNA) is its high mutation rate due to which has accumulated several polymorphisms, temporal and geographically structured, reflecting the prehistoric spread of humans from Africa to the rest of the world [8]. These diagnostic polymorphisms have been used to assort related individual mtDNA types (haplotypes) into groups (haplogroups) following a hierarchical nomenclature. Major haplogroups were denoted by uppercase roman letters and successive nested groups by alternating integers and lowercase roman letters [9]. For instance, all mtDNA haplotypes of African origin belong to a macro-haplogroup named “L” that comprises several related haplogroups named L0, L1, L2, L3, each of which was further subdivided into sub-haplogroups as L0a, L0b, L0c, and so on. It has been demonstrated that different haplogroup backgrounds could modify numerous disease predispositions [10]. Recently, it has been reported that African mtDNA haplotypes belonging to L haplogroups, excluding L2, confer susceptibility to POAG in the Saudi Arabian population [11]. Taking into account the fact that prevalence of POAG in Africans and populations of African descent is significantly higher than in Europeans and Asians [12], this report raised the possibility that some mtDNA African haplogroups were, at least in part, responsible for the susceptibility of Africans to POAG. To test this hypothesis, we compared mtDNA Haplogroups frequencies in POAG patients from Ghana with that of ethnicity matching controls.

## Methods

### Patients and control subjects

We recruited 373 Ghanaian POAG patients (with a mean age of 62±12) who satisfied strict clinical criteria for POAG which includes the following: i) appearance of the disc or retinal nerve fiber layer e.g., thinning or notching of disc rim, progressive changes, nerve fiber layer defect; ii) the presence of characteristic abnormalities in visual field (e.g., arcuate scotoma, nasal step, paracentral scotoma, generalized depression) in the absence of other causes or explanation; iii) age greater than 40 years, and iv) open anterior chamber angles bilaterally on gonioscopy. Exclusion criteria included evidence of secondary glaucoma, e.g., pigmentary dispersion syndrome, pseudoexfoliation, history of steroid use or ocular trauma. All cases had onset of glaucoma after age 40 (adult-onset POAG). Patients were recruited from the glaucoma clinic in Accra after signing an informed consent approved by the institutional review board.

A second group (n=451 with a mean age of 53.2±10 years) of healthy Ghanian controls free from glaucoma by examination were recruited. Entry criteria for those subjects were age >40, normal IOP, open angles on gonioscopy, and normal optic nerves on examination.

### DNA extraction

Peripheral blood (5 ml) was collected in EDTA tubes from all participating individuals. DNA was extracted using the illustra blood genomicPrep Mini Spin Kit from GE Healthcare (Buckinhamshire, UK), and stored at –20 °C in aliquots until required.

### Mitochondrial haplogroup assortment

All samples were polymerase chain reaction (PCR) amplified and sequenced for the mtDNA regulatory region hyper variable segment I (HVS-I) and, when necessary, for coding segments comprising one or more additional diagnostic positions (Table 1). Previously described primers and conditions [13] (Table 2) were used. Each 25 µl PCR reaction contained 2.5 µl of 10× reaction buffer with MgCl_2_ (Amersham Pharmacia Biotech, Piscataway, NJ), 10 pmol of each primer, 100 pmol/µl each of deoxynucleoside triphophates (deoxyadenosine triphosphate, deoxyguanosine triphosphate, deoxycytidine triphosphate and deoxythymidine triphosphate; Perkin-Elmer Corporation, Foster City, CA) in Tris HCl buffer, 1 unit Taq DNA polymerase (Amersham Pharmacia Biotech) and 50 ng genomic DNA template. The mixture was denatured at 95 °C for 5 min and the PCR reaction was carried out for 35 cycles, in a GeneAmp 9700 PCR system (Perkin-Elmer Corporation), under the following conditions: denaturation at 95 °C for 1 min, annealing at 54 °C for 45 s, extension at 72 °C for 1 min and final extension cycle of 72 °C for 7 min. The PCR products were electrophoresed on a 1% agarose gel and detected with 0.5 µg/ml ethidium bromide to confirm the correct amplicon size. Composite haplotypes were assorted into haplogroups following the most recent mtDNA haplogroup nomenclature [14].

**Table 1:** Haplogroup distribution in POAG patients and controls.

** **	**Control (n=451)**		**Case (n=373)**		** **	**Confidence interval**		** **
**Haplogroup**	**N**	**%**	**N**	**%**	**Odds ratio**	**Lower limit**	**Upper limit**	**Fisher test p-value**
L	0	0	1	0.3	-	-	-	0.45
L0	1	0.2	0	0	-	-	-	1.00
L0a	11	2.4	11	2.9	1.22	0.52	2.84	0.67
L1b	33	7.3	36	9.7	1.35	0.83	2.22	0.26
L1c	31	6.9	23	6.2	0.89	0.51	1.56	0.78
L2	17	3.8	15	4.0	1.07	0.53	2.17	0.86
L2a	147	32.6	118	31.6	0.96	0.71	1.28	0.82
L2b	27	6.0	12	3.2	0.52	0.26	1.05	0.07
L2c	10	2.2	8	2.1	0.97	0.38	2.47	1.00
L2d	3	0.7	0	0	-	-	-	0.26
L2e	3	0.7	1	0.3	0.40	0.04	3.88	0.63
L3	6	1.3	1	0.3	0.20	0.02	1.66	0.14
L3a	1	0.2	3	0.8	3.65	0.38	35.22	0.33
L3b	33	7.3	36	9.7	1.35	0.83	2.22	0.26
L3d	43	9.5	24	6.4	0.65	0.39	1.10	0.12
L3e	70	15.5	62	16.6	1.09	0.75	1.58	0.70
L3f	8	1.8	13	3.5	2.00	0.82	4.88	0.13
L3h	0	0	1	0.3	-	-	-	0.45
L4	2	0.4	2	0.5	1.21	0.17	8.63	1.00
L4b	1	0.2	0	0	-	-	-	1.00
M1	0	0	1	0.3	-	-	-	0.45
U6a	4	0.9	5	1.3	1.52	0.40	5.70	0.74

**Table 2:** Primers used for mitochondrial haplogrouping.

**Primer #**	**Primer sequence**	**Annealing temp.**	**Product size**
MIT-1-F	CCTCCCTGTACGAAAGGACA	57	614
MIT-1-R	TGAGATTGTTTGGGCTACTGC		
MIT-2-F	CGAGCAGTAGCCCAAACAAT	57	664
MIT-2-R	TTTTGGATTCTCAGGGATGG		
MIT-3-F	CCATCCCTGAGAATCCAAAA	57	653
MIT-3-R	ATTTTGCGTAGCTGGGTCTG		
MIT-4-F	TAAACCAGACCCAGCTACGC	55	661
MIT-4-R	AAAGTGGCTGATTTGCGTTC		
MIT-5-F	TCAACTGAACGCAAATCAGC	60	784
MIT-5-R	TGAAATTGATGGCCCCTAAG		
MIT-6-F	CTTAGGGGCCATCAATTTCA	57	705
MIT-6-R	AAGCCTCCTATGATGGCAAA		
MIT-7-F	GCCATCATAGGAGGCTTCATT	59	702
MIT-7-R	TTTCCTGAGCGTCTGAGATGT		
MIT-8-F	CATCTCAGACGCTCAGGAAA	55	730
MIT-8-R	GGGAGGTAGGTGGTAGTTTGTG		
MIT-9-F	TACTACCGTATGGCCCACCA	60	781
MIT-9-R	AGGCTTGGATTAAGGCGACA		
MIT-10-F	ACTGACTATCCTAGAAATCGCTGT	59	741
MIT-10-R	GCAGATAGTGAGGAAAGTTGAGC		
MIT-11-F	CCACGGACTTCACGTCATTA	59	743
MIT-11-R	GGGAGGATATGAGGTGTGAGC		
MIT-12-F	GCATTTACCATCTCACTTCTAGG	57	751
MIT-12-R	AGTGCGATGAGTAGGGGAAG		
MIT-13-F	AGGCACATACTTCCTATTCTACACC	55	740
MIT-13-R	TGATATTTGATCAGGAGAACGTG		
MIT-14-F	ACTGGGAGAACTCTCTGTGCT	55	740
MIT-14-R	ACGAACAATGCTACAGGGATG		
MIT-15-F	TCCATAATATTCATCCCTGTAGCA	57	741
MIT-15-R	GTGGGTACAGATGTGCAGGA		
MIT-16-F	GTTACAATCGGCATCAACCA	55	740
MIT-16-R	AGCTTTTCTAGTCAGGTTAGGTCTA		
MIT-17-F	CCTCAACCCAAAAAGGCATA	59	745
MIT-17-R	GGAGGTCGATGAATGAGTGG		
MIT-18-F	ACGCAAAATTAACCCCCTAA	59	742
MIT-18-R	GCCTAGGAGGTCTGGTGAGA		
MIT-19-F	CCTAGGCGACCCAGACAAT	59	740
MIT-19-R	GATAGTTGAGGGTTGATTGCTGTA		
MIT-20-F	TGCTTACAAGCAAGTACAGCAAT	57	370
MIT-20-R	TGATGTCTTATTTAAGGGGAACG		
MIT-21-F	GGCTCACATCACCCCATAAA	55	720
MIT-21-R	CATGGGCTACACCTTGACCT		
MIT-22-F	GCAAACCCTGATGAAGGCTA	57	632
MIT-22-R	GGGGTCTTAGCTTTGGCTCT		
MIT-23-F	ACTTTGCAAGGAGAGCCAAA	59	672
MIT-23-R	AGGCGGTGCCTCTAATACTG		
MIT-24-F	TCACCTCTAGCATCACCAGTATT	57	660
MIT-24-R	GGAAGGCGCTTTGTGAAGTA		
HVS-I-F	ACT TCA CAA CAA TCC TAA TCC T	60	1200
HVS-I-R	CGG AGC GAG GAG AGT AGC AC		
HVS-II-F	CAT TTA CCG TAC ATA GCA CA	60	820
HVS-II-R	GGG AGG GGG TGA TCT AAA AC		

### Data analysis

The frequency of each haplogroup among cases and controls were compared with the Fisher’s exact test, and the risk of having the disease if a subject have a certain haplogroup as compared to not having that specific haplogroup was estimated by computing odds ratio and its 95% confidence interval. Mean age of males was compared with the females mean age by *t*-test. A p-value less than 0.05 was considered significant. There was no need to adjust the significance level of a statistical test to protect against Type I errors when multiple comparisons were being made because we did not find any significant association between any of the mitochondrial haplogroups and the outcome. All analyses were performed using SPSS v.13 statistical analysis software (SPSS Inc., Chicago, IL).

## Results

Our cohort consisted of 373 POAG Ghanaian-patients and 451 controls from Accra and Tema in the south of Ghana. There was a statistical significant difference in age (p=0.0001) between patients (mean age 62±12 years) and controls (mean age 53.2±10 years). Table 1 shows the mitochondrial haplogroup distribution among both groups. The results presented in Table 1, shows that there was no statistically significant difference between patients and controls for all mitochondrial haplogroups tested.

## Discussion

A well known risk factor for POAG is African descent. At least in part, it seems correlated with other risk factors more prevalent in Africans than in Europeans as thinner corneas, higher percentage of reported diabetes mellitus, or high blood pressure [15]. In addition, it has been reported that mtDNA African L haplogroups, excluding L2, confer susceptibility to POAG in the Saudi Arabia population [11].

We planned to analyze mtDNA frequencies in an African population with high prevalence of POAG, not directly related to the Saudi population and without important ethnic structuring for mtDNA haplogroup frequencies and POAG prevalence. It seemed us that the Ghana population fulfilled these requisites because it has prevalence of POAG comparable to those in African descent populations of USA, Barbados or St. Lucia [16]; it is located in West Africa, far away of the Arabian peninsula and showed a lack of difference in prevalence among different ethnic groups [16] and a great homogeneity of mtDNA profiles in this and surrounding west African countries across different linguistic communities [17,18]. Consequently, the main aim of this work was to compare the mtDNA haplogroup frequencies between cohorts of POAG patients and their age-matched controls in general.

Recently, Fendt et al. [17] performed mtDNA haplogrouping on 191 Ghanaian subjects. Whereas the cohort analyzed in this paper are derived from the Ghanaian coastal cities of Accra and Tema which contain multiple tribal groups, that analyzed by Fendt, et al. [17] were primarily from the Akan tribal group in the northern region of the country. The prevalence of most major mtDNA haplogroups was significantly different from that in our control population. Due to these population differences, an analysis comparing cases and controls in our study was not possible.

We compared the mitochondrial haplogroups distribution among POAG patients from Accra and Tema with controls from the same area. The results indicate that there was no statistically significant difference between patients and controls for all mitochondrial haplogroups tested. Thus, at least in this part of Ghana, it seems that mtDNA haplogroups do not play a role for the development of POAG. Accra and Tema are located in the deep south of Ghana at the gulf of Guinea coastline. The sub-Saharan African mtDNA haplroup L2 had similar frequencies in both POAG patients and controls and the little difference in frequency was not statistically significant (p=0.86).
